# Design Path for a Social Robot for Emotional Communication for Children with Autism Spectrum Disorder (ASD)

**DOI:** 10.3390/s23115291

**Published:** 2023-06-02

**Authors:** Sandra Cano, Jaime Díaz-Arancibia, Jeferson Arango-López, Julia Elena Libreros, Matías García

**Affiliations:** 1 School of Computer Engineering, Pontificia Universidad Católica de Valparaíso, Valparaíso 2340000, Chile; matias.garcia@pucv.cl; 2 Departamento de Ciencias de la Computación e Informática, Universidad de la Frontera, Temuco 4811230, Chile; jaimeignacio.diaz@ufrontera.cl; 3 Departamento de Sistemas e Informática, Universidad de Caldas, Manizales 170004, Colombia; jeferson.arango@ucaldas.edu.co; 4 Facultad de Psicología, Universidad Cooperativa de Colombia, Cali 760035, Colombia; julia.libreros@campusucc.edu.co

**Keywords:** human–robot interaction, autism spectrum disorder, user-centered design

## Abstract

Children with autism spectrum disorder (ASD) have deficits in social interaction and expressing and understanding emotions. Based on this, robots for children with ASD have been proposed. However, few studies have been conducted about how to design a social robot for children with ASD. Non-experimental studies have been carried out to evaluate social robots; however, the general methodology that should be used to design a social robot is not clear. This study proposes a design path for a social robot for emotional communication for children with ASD following a user-centered design approach. This design path was applied to a case study and evaluated by a group of experts in psychology, human–robot interaction, and human–computer interaction from Chile and Colombia, as well as parents of children with ASD. Our results show that following the proposed design path for a social robot to communicate emotions for children with ASD is favorable.

## 1. Introduction

Autism spectrum disorder (ASD) is considered by both the World Health Organization (WHO) and the Diagnostic and Statistical Manual of Mental Disorders (DSM-5) as a condition characterized by deficits in two domains: (1) social communication and social interaction and (2) restricted repetitive patterns of behavior, interest, and activities. ASD is considered to have a wide variety of clinical and behavioral expressions, resulting from multifactorial dysfunctions in the development of the central nervous system [1]. In Latin America, ASD diagnoses have increased. In Chile, ASD’s clinical practice guide for detection and diagnosis, published in 2011 by the Ministry of Health, estimated the prevalence at 89.6 cases per 10,000 newborns in 2007. Therefore, developed countries present two new cases yearly for every 500–700 newborns [2]. In turn, most research into autism spectrum disorder has been conducted in affluent English-speaking countries [3]. Therefore, there has been a notable increase in the use of technology that can support therapies, mainly focused on emotional communication. In addition, the recognition of autism in girls is a challenge, as most diagnostic tools have been developed primarily based on the observation of boys’ behaviors [4]. This results in ignored cases of ASD in girls who simply do not show the same behaviors observed in boys. Without diagnosis for these girls, the relative proportion of boys with autism will increase.

Social-assistive robots (SARs) have attracted increasing interest in healthcare and therapy, especially for children with ASD. In these children, interaction and communication with SARs has been found to improve their collective attention capacity, ability to recognize emotions, and development of social competence, among other benefits [5]. Children with ASD typically do not see these robots as mechatronic mechanisms running via a computer program. Instead, they assign characteristics that are expected to be attributed to living systems [6], and studies have found that children with ASD respond positively to robots [7]. Therefore, interaction with robots may be a better alternative for children with ASD to learn to perceive, understand, and express emotions, as opposed to traditional therapy.

Many social robots have been proposed, such as NAO [8], KASPAR [9], Moxie [10], QTrobot [11], and AIBO [12]. However, few studies have been conducted on how to design a social robot for children with ASD. Non-experimental studies have been conducted to evaluate social robots, yet a methodology for design is rare. A study by Kunold et al. [13] proposed a framework to study and design communication with social robots based on Laswell’s 5Ws of mass communication: who says what, in which channel, to whom, and with what effect. The authors extended this model to communication in human–robot interaction. In another study, Su and Shuzhi [14] proposed a methodology for designing the appearance and interaction methods of social robots. The authors considered three core variables of social attributes:

1. Situations include questions such as the native language and cognition of the robot, culture, and scenes.
2. Objects include who, how, and goal.
3. Roles include gender, bio-sociological, social differentiation, and cultural situation roles.

Bartneck and Forlizzi [15] proposed a design-centered framework for social human–robot interaction. The framework contains the following properties: form, modality, social norms, autonomy, and interactivity. In 2021, Axelsson et al. [16] proposed a framework to design social robots based on a set of canvas tools for participatory design, which is composed of the following three phases of the design process: (1) the definition of the problem space; (2) the creation of design guidelines; and (3) the integration of these guidelines in the solution space. Another case study [17] applied participatory design in the creation of a social robot for autism, where the authors proposed the following set of stages: (1) sensitization; (2) focus group with stakeholders; (3) generative intervention with children; (4) validation and ratification of the preliminary findings; (5) perceptual maps and conceptual design; (6) preliminary 2D/3D prototyping with community feedback; (7) detailed design and manufacturing; and (8) results.

Another study proposed guidelines for designing social robots as second language tutors [18], including (1) age differences; (2) target word selection; (3) the use of a meaningful context and interactions to actively involve children; and (4) the dosage of the intervention. Bradwell et al. [19] provided design recommendations for social robots in health and social care. The studies reviewed indicate a lack of design tools, guidelines, and methodologies to facilitate the design of a social robot for emotional communication, especially for children with ASD. Therefore, the research question guiding this work is as follows: How can we design a social robot for emotional communication for children with ASD?

The remainder of this paper is structured as follows: Section 2 provides a brief description of the concepts related to this work. Section 3 proposes a design path for creating a social robot for children with ASD and describes each of its component aspects. Section 4 presents a case study following the proposed design path, and Section 5 includes a discussion. Finally, conclusions are presented in Section 6.

## 2. Background

### 2.1. Communication Strategies with ASD

Some activities that have been found as communication strategies in children with ASD are as follows: (1) *Imitation,* which plays a significant role in the transfer of knowledge to the child from an external source, where the child learns new physical and verbal skills and explores his or her social existence [20]. (2) *Eye contact*, which has been shown to be more useful than verbal communication. In addition, eye contact serves not only to monitor each other’s state of attention and emotion but also to establish mutual acknowledgment. (3) *Joint attention*, or the act of sharing attentional focus [21,22], which is defined as two individuals looking at the same target through eye gaze or pointing by means of hand gestures. (4) *Turn-taking*, where children with autism find it extremely difficult to share things and indulge in normal conversations involving taking turns with others [23]. (5) *Emotion recognition and expression*, where children with ASD find it very hard to read and interpret facial expressions and body language. Interactions with others can involve excessive sensory stimulation, causing severe distress to children with autism [24]. (6) *Self-initiated interactions*, which are related to difficulties in asking for things which autistic children need.

### 2.2. Social Robots for ASD

A social robot is a robotic platform that integrates a computational model able to communicate and interact, understand, and even relate to us in personal way [20]. However, designing a social robot for ASD is a challenge, especially if the interaction is between a child and robot, as children have limitations with respect to their perceptual, motor, and cognitive abilities compared with adults. The first social robot with emotional abilities was Kismet [25]. Kismet was designed with a mechanism to help it cope with a complex social environment, where many humans communicate emotional expressions using their face. This social robot can express emotional states such as happiness, sadness, surprise, anger, calm, displeasure, fear, interest, and boredom. This expression of emotions is achieved through the face, voice, and movements.

The first robot conceived for therapy for children with ASD was from the AURORA project [26]. Later, Robota [27] was created, a doll with five degrees of freedom (DoFs) that could move its head, arms, and legs. This robot reacted to touch and used voice synthesizers to attract the attention of children with ASD. Then, a robot with a humanoid appearance and 29 DoFs appeared, named Infanoid [28]. Cameras were placed in both eyes of the robot for real-time detection, where the eyes could perform saccades and smoothly track a visual target, while movement in its eyes, lips, and eyebrows allowed it to express different emotional states. Infanoid also had microphones located in each of its ears, which allowed it to capture and analyze sounds.

In 2005, Kaspar, with a more human and childlike appearance, was developed and used for therapeutic purposes in children with ASD [29]. Kaspar was designed with 16 DoFs and measured approximately 46 cm tall. The behavior of the robot was controlled remotely by a human operator. It contained cameras for detecting objects. This first version of Kaspar contained servomotors located in the face to make facial expressions. As such, it had three DoFs in each eye/eyelid, two in the mouth, three in the neck, five in each arm, and one in the torso. Another robot design is Keepon [30], whose appearance is non-, with a yellow snowman-like body that is 120 mm tall. It has color cameras in both eyes and a nose that acts as a microphone, along with a silicone body with four DoFs. Keepon can turn its head up/down and left/right for eye contact. It expresses its emotions through body movements. For example, it sways from left to right or up/down. It also accentuates brief sounds from a built-in speaker. Keepon can express what it perceives.

Several robots have been proposed exclusively for children with ASD, such as KASPAR [31], Leka [32], QTrobot [11], Milo [33], Buddy [34], Castor [35], and Moxie [10]. Most of them are humanoid in appearance, while Leka is a multisensory spherical robot that emits subtle vibrations, lights up with colorful LEDs, plays music, and chirps in an anthropomorphic fashion. There are also zoomorphic robots such as Probo [35], Pleo [36] and Romibo [37]. In addition, the majority of social robots proposed use the English language and only Castor is Latino; this indicates that most of these robots are not designed for a Latino society, where culture is still a factor that affects an intervention. Therefore, the design of robots for children with ASD is still unclear with respect to their ideal appearance, since both anthropomorphic and non-anthropomorphic robots are found in the literature.

### 2.3. Emotional Communication

Bartsch and Hübner [38] proposed a framework for emotional communication that comprises three interrelated levels of complexity: (1) innate stimulus–response patterns, (2) associative schemata, and (3) symbolic meaning. This suggests that people not only communicate to exchange information but also to exchange emotions. Therefore, emotional communication can be defined as a process of mutual influence between the emotions of communication partners. Reis and Sprecher [39] define emotional communication as the process of using messages to exchange information about and influence each other’s emotional states, where messages may be verbal and nonverbal expressions of emotion. Derks et al. [40] define emotional communication as the recognition, expression, and sharing of emotions or moods between two or more individuals, which includes both explicit and implicit emotional communication. Huang and Rust. [41] proposed four intelligences that can be employed by machines: mechanical, analytical, intuitive, and empathetic. Empathetic intelligence is the ability to recognize and understand other people’s emotions, respond appropriately emotionally, and influence other’s emotions. Empathetic AI is the most advanced generation of artificial intelligence, where empathetic tasks are social, emotional, communicative, interactive, and relational.

Emotional communication is related to emotion theories and communication theories. There is no single definition or model. Theories of emotion can be grouped into three approaches: physiological, neurological, and cognitive. Williams Lange’s theory [42] proposes a physiological approach, contending that emotions occur as a result of physiological reactions to events. Shachter and Singer [43], from a cognitive approach, propose that emotions are composed of two factors, physiological and cognitive, i.e., a stimulus leads to a physiological response that is then cognitively interpreted and labeled [44]. Meanwhile, Damasio proposes a neurobiological approach, which includes Damasio’s somatic marker [45], in which he explains the relationship between emotions and reason.

Theories of communication provide a way to exchange information between one or more people. Shannon [46] proposed a mathematical theory of communication that consists of five parts: (1) *an information* source that produces a message, where various combinations can also occur, for example, a visual channel associated with an audio channel; (2) *the transmitter,* which operates on the message in some way to produce a signal suitable for transmission over the channel; (3) *the channel,* which is the medium used to transmit the signal from the transmitter to receiver; (4) *the receiver,* which performs the inverse operation of that performed by the transmitter; (5) *the destination,* which is the person for whom the message is intended. Lasswell [47] describes an act of communication as answering the following questions: who says what, in which channel, to whom, and with what effect?

### 2.4. Social Robots’ Emotional Communication

Social robots can capture information about the environment through sensors. Therefore, a robot can have several channels to capture information, including visual, auditory, physiological, and tactile. This can provide information about emotions transmitted by a person, which can be manifested as verbal and non-verbal (gestures) expressions. The social robot receives the information (message) and interprets and expresses an emotional behavior. Thus, for communication between humans, the robot represents a process that includes a transmitter, message, and receiver, where messages are interchanged through communication channels that allow input and output.

The input describes how information is captured by the robot, while the output describes how the robot expresses itself to the human. Both the input and outputs can include visual, auditory, and tactile channels. However, the physiological response is rarely considered. In addition, designers of social robots should take all perceivable cues of a robot, as well as the human audience, into account when estimating communication effects, e.g., visible cables and emergency stop, or the color of the LED lights [48].

A study by Bonarini [49] reported that all signals involved in the different channels were coherent, in order to obtain effective message exchange and establish a good relationship between the human and robot. However, the study mentioned limitations imposed by sensors, computational power, mechanical implementation, and the role to be played.

Transmitting information is essential so the user can motivate and understand the message. One study [50] found that individuals with ASD often show superior visual detection. Moreover, they often exhibit atypical sensory behaviors, such as adverse responses to specific sounds or textures, object touching, and visual fascination with lights or movement.

#### 2.4.1. Visual Channel

Robots can exploit the visual channel through light, images, or motion. In addition, they can also perceive information from the environment through sensors, such as cameras. For example, the robot may detect an object through a sensor, which prevents it from moving in a certain direction; its reaction may be to choose another path or to keep still. If the robot is to have cognitive behavior, support from computational models is needed to allow the robot to predict motor intentions.

Light can be emitted by LEDs or other light sources that exploit color, intensity, and rhythm to express a message or emotion. These LEDs can be organized into matrices, which can be used on the eyes and mouth, in addition to other areas.

The screen is used to animate parts of the faces and can also be used to interact with users through the robot to allow for telepresence. Another alternative is motion, with which the robot can express a behavior with its body or parts of it. In addition, this is used for facial expressions because they are key to understanding emotions [51].

On the other hand, robots can capture information through the visual channel environment using sensors such as cameras or infrared thermal cameras, which can detect emotional changes through variations in skin temperature. Usually, this is achieved by considering temperature variations in specific regions such as the nose, forehead, and cheeks [52].

#### 2.4.2. Hearing Channel

The hearing channel enables the robot to capture sounds from the environment. The sensors used to capture sound come with one or more microphones and are usually located in each ear of the robot. There is also the case of ultrasound sensors, whose purpose is to measure the distance of ultrasonic waves to detect objects. Thus, this type of sensor emits and receives ultrasonic waves to measure the distance from an object; its operation is, therefore, as a proximity sensor. Usually, these types of sensors are found in educational robots. Due to their shape, they can simulate the robot’s eyes. Meanwhile, the microphones can simulate the ears localized on each side of the head; an example is the Nao robot.

On the other hand, sounds can be used by robots to express emotions. However, speech with children with ASD can be difficult when the individual with ASD has difficulty conversing. A study by Barakova et al. [53] examined the number of vocal interactions between child and robot, which they found did not significantly increase across sessions.

A hearing channel is a form of verbal communication that uses computer models to perceive/recognize sounds. As such, it can follow instructions through speech [54]. For this type of verbal communication, the support of natural language processing techniques is needed so that the robot can verbally understand the user [55].

Another alternative in low-cost robots is to use a sensor to emit sounds recorded in mp3 format to execute voice commands, along with speakers to produce the sounds. This can be integrated with natural language computational models so the robot responds verbally. Therefore, some researchers have focused on a question-and-answer dialogue.

#### 2.4.3. Touch Channel

The touch channel is used in humans to perceive and interpret stimuli from the environment. This perception is achieved in robots using touch sensors with different purposes, either to measure forces in a particular area, or as contact. However, this tactile perception occurs at a single point of contact, acting as an artificial skin [56]. For example, the Nao robot has a single point of contact, a touch sensor located on the top of the head [57]. In robotics, tactile information is used as a control parameter [58]. One of the most straightforward sensors to interact in a tactile way is the pulsed sensor, which is represented by a binary state (1, pulsed; 0, not pulsed). It can be used for shock control and to detect the presence of an object. Furthermore, there are capacitive sensors, which are often used to detect affective gestures such as hugs or caresses [59]. The touch channel has become a key technology for interaction with robots, either for sensing or vibro-tactile response [60].

Some robots are equipped with accelerometer and gyroscope sensors that are used to detect a wide range of activity, usually by an implicit communication. Data capture through inertial sensors (accelerometer and gyroscope) can be used to interpret manipulations or gestures [61].

#### 2.4.4. Physiological Channel

Sensors that capture the physiological responses of humans have been repurposed for emotional detection, where physiological sensors for temperature, pulse, galvanic response, etc., have been used. However, as already mentioned, children with ASD are not aware that their emotions can affect their physiological responses. For example, being afraid is associated with sweating (galvanic response), an increased heart rate (faster heartbeat), and high levels of adrenaline, which make us extremely alert [62]. Depending on the context, this experience of fear can be positive or negative. Furthermore, when angry, our body temperature, heart rate, and blood pressure all increase [63]. In some people, due to the change in temperature, the blood vessels dilate, causing the ears to change to a red color; similarly, the nose becomes red when cold. However, these types of expression are usually involuntary reactions and are often difficult to regulate.

## 3. Design Path

Designing social robots requires a deep understanding of human behaviors, especially when considering children with ASD. The design of social robots is multidisciplinary because it includes areas such as psychology, neuroscience, human factors, design, anthropology, and artificial intelligence, among others. Therefore, we propose a path of design based on the user-centered design (UCD) approach [64] to create a social robot for individuals with ASD. This proposal was mainly influenced by two studies conducted by the authors of [16,65,66].

### 3.1. Analysis

To determine a problem’s solution, it is necessary to analyze the use context and users, focusing on what characterizes the user and what they are trying to achieve, i.e., their needs. Here, the participants are parents, therapists, and children with ASD. Methods such as interviews, questionnaires, focus groups, and direct observation can be used to investigate participant’s perceptions. In one study [17], sensitization was used to collect views and ideas on and about robotics, where the authors formulated three questions: how participants imagined a robotic device, how robots could assist in therapies, and how participants imagined robots could provide benefits in therapy.

Children with ASD have difficulties with communication and social interaction. Therefore, tools that allow for the collection of information from children with non-verbal communication through emotion analysis techniques are useful. However, the analysis of emotions requires experts. Empathy map (EM) is one technique used in the design thinking methodology. EM can be used by inexperienced developers and/or designers to provide short-term results. Melo et al. [66] proposed an empathy map centered for autism called EmpathyAut based on the traditional empathy map model. EmpathyAut covers the areas, such as cognition/learning (feel/think), interaction (see), communication (say), behavior (do), relationship with sound/noise level (listen), needs (pain), and motivations (gain). The authors designed a characterization form that included 33 items representing the absence (0) or presence (1) of the characteristic in an autistic person. EmpathyAut is aimed at children with ASD, especially those who are low/medium functioning.

In one study, focus groups, which represent a quality method, were used with parents and professionals to identify intervention requirements for creating a new robot [66]. The activities included (1) capturing demographic information; (2) a demonstration of a social robot called Kaspar; and (3) a discussion of how interventions with the Kaspar robot should be carried out. Other techniques such as direct observation, interviews, and questionnaires have also been used to collect information from parents of children with ASD.

Comparative studies, which allow for the analysis of three or more robotics platforms, comparing their functionalities and aspects and distinguishing their similarities and differences, have also been carried out. A study conducted by Puglisi et al. [67] involved the selection of a set of social humanoid commercial robots for children with ASD for which a comparative analysis was carried out, identifying the characteristics, advantages, and disadvantages of each robotic platform.

### 3.2. Ethical Considerations

Ethical considerations related to the relationship between the user and robot should guide the interactions between them [68]; some aspects that should be considered include physical safety, data security, transparency, emotional consideration, and behavior. Value-sensitive design is the integration of ethics during the technology’s design that accounts for human values in a principled and comprehensive manner throughout the design process [69].

### 3.3. Aspects of Design

The aspects that must be considered are the environment, form, modality, communication, and behavior.

#### 3.3.1. Environment

The environment examines the role of the robot in interacting with children with ASD. Some studies have reviewed the interaction between children with ASD and social robots. Huijnen et al. [65] defined seven roles for this relationship: provoker, reinforcer, trainer, mediator, prompter, diagnostician, and buddy. We defined robot roles such as therapist, assistant, mediator, and play partner.

#### 3.3.2. Form

Form, which refers to the appearance of a robot for children with ASD, is an important variable influencing the interaction between the child and robot. However, some studies have reviewed anthropomorphic and caricatured robots. In the literature, one study found that a humanoid robot was perceived as humanlike and elicited stronger expectations about the robot’s social and cognitive competencies [70] than a zoomorphic robot, which was perceived as having more the functions of an animal, along with a lower level of functioning. In addition, if the robot is humanoid, it should be considered whether it is a boy/girl or adult. The studies reviewed show that most of the robots designed have the appearance of a child. In addition, the size of the robot can affect the interaction, especially in children with ASD working on attention or joint attention [71], where the robot must establish visual contact with the child. The movements of a robot can be rated from machine-like, to hybrid, to life-like. However, smooth movements require more mechanical movement quality, which can be used to express emotions and maintain social relationships.

#### 3.3.3. Modality

Modality is defined as the number of communication channels engaged, which can vary from unimodal (one channel) to multimodal. This aspect considers visual, auditory, haptic, and physiological channels.

#### 3.3.4. Communication

Communication is related to communication channels, which can be visual, auditory, touch, and physiological. In addition, the language must be considered, which can influence social cues between humans and robots.

#### 3.3.5. Interaction

Interaction refers to the autonomy of the operation mode of the robot, which is in turn related to its capabilities to act without direct input from a person. Autonomy is a requirement for social robots. A remote-controlled robot cannot be considered social, since it does not make decisions by itself.

#### 3.3.6. Intelligence

This aspect is related to the work proposed by Huang and Rust [41], who proposed artificial intelligence levels that can be manifested by machines as a service. These artificial levels are mechanical, analytical, intuitive, and empathetic. Mechanical AI learns or adapts at the minimum; analytical AI learns and adapts systematically based on data; intuitive AI learns and adapts intuitively based on understanding; and empathetic AI learns and adapts empathetically based on experience.

#### 3.3.7. Behavior

One study [72] carried out a comparison of several robots for use in therapy for children with autism, grouping different robots with respect to therapy model and targeted behavior. Behavior was defined as a set of skills, such as imitation, joint attention, turn-taking, self-initiated interactions, and expressing emotions.

### 3.4. Ideas of Design

There are several tools and methods that can be used to support the generation of ideas. Cards are a very common tool in design workshops that are used to support participatory design activities [16]. The cards can be used to build new ideas and guide the design process. These tools can also be utilized to identify requirements in the design of a social robot. In this stage, it is important to consider the guidelines used in human–robot interaction (HRI) to create robotic applications. These include guidelines for robot behaviors [73], guidelines for designing a social robot for autism [17], and general HRI guidelines [74].

### 3.5. Prototypes

In software prototyping, low-fidelity scenarios and wireframes can be used during the early requirement analysis. Low-fidelity wireframes are usually based on drawings of the 2D interfaces of objects, such as menus, buttons, etc. Therefore, when applied to robots, the robotic platform is the interface, which considers aspects such as form, modality, and communication. In addition, in robotic prototyping, it is important to make the scenarios interactive; this can be implemented using storytelling related to the flow of interaction, which can be analyzed using aspects such as interaction and behavior.

## 4. Case Study

In this case study, the design path proposed here to create a social robot for children with ASD was followed.

### 4.1. Analysis

**Figure 1:**
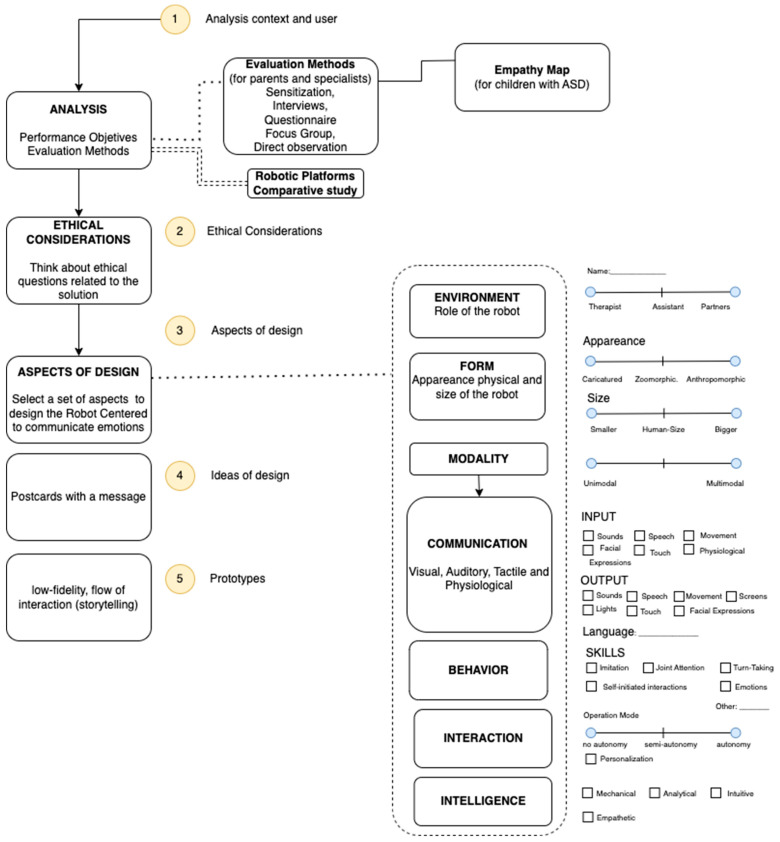
Path for designing a social robot for children with ASD.

Since children with ASD have communication problems, panels composed of experts, therapists, parents, and teachers were asked to give their opinion. To determine the needs of children with ASD, we designed a questionnaire that was administered to 18 parents of children with ASD and five specialists (psychologist, neuropsychologist, nutritionist, and social worker) from Chile. The questionnaire was composed of two parts. The first part was designed to collect information about the child’s emotional state (express, perceive, and understand), kinds of intervention therapies, and use of technology in therapy. The second part involved the assignment of scores on a five-point Likert scale to a set of robot cards in order to determine the children’s appearance preferences. Ten references to robots were selected considering appearance, including anthropomorphic, biomimetic, and non-biomimetic (see Figure 1).

The questions given to and answers provided by the specialists are outlined below.

**Q1: *What types of therapy have been used for children with ASD?***

RQ1: Floor-time/RDI, for the development of social skills; executive function and analytical therapy; behavioral-based therapy; nutritional approaches; and socio-affective therapy.

**Q2: *What strategies have been used to teach children with ASD to understand that emotions can be associated with physiological signals?***

RQ2: Multimedia content (videos), drawings, visual aids and phonological gestures (AAC), self-knowledge, role-playing, speaking, etc.

**Q3: *Have you used technologies in therapy interventions?***

RQ3: 60% answered “yes”, 40% answered “no”.

**Q4: *Imagine therapy with robots—what would be the best and worst aspects?***

RQ4: It should not be too colorful; it should be gentle and soft. The child’s attention should be held, and they should feel at ease and curious. It should look friendly and have pleasant characteristics such as, for example, a pleasant smell, sweet voice, neutral texture, etc. Stimuli should be limited.

**Q5: *If a person with ASD interacts with a robot, what kind of physical communication gestures should the robot make?***

RQ5: It should be trained in basic emotions, with a prosody that is not particular but very natural, speaking at a decibel level in accordance with the hyper-sensitivity of some children with ASD. It should have a very human face. Another response suggested that the robot would perhaps make a pattern, and with this it would most likely discover something that we as humans do not recognize. In addition, the robot must offer greetings and smile and respond to the child’s gestures.

The robot should look directly into the child’s eyes, which is the most difficult thing for children with ASD, always facing him/her, not too close, with relaxed hands or arms that they can see, so that they do not feel fear or other negative emotions. The robot must keep its distance and let the child touch it in order to build trust.

**Q6: *What kind of affective tactile communication should the robot be able to feel, and what part of its body should be sensitive to touch?***

RQ6: The robot should be sensitive to the child’s head, face, and eyes. The parts of the body that should be sensitive to touch include the hands, head, and abdomen.

**Figure 2:**
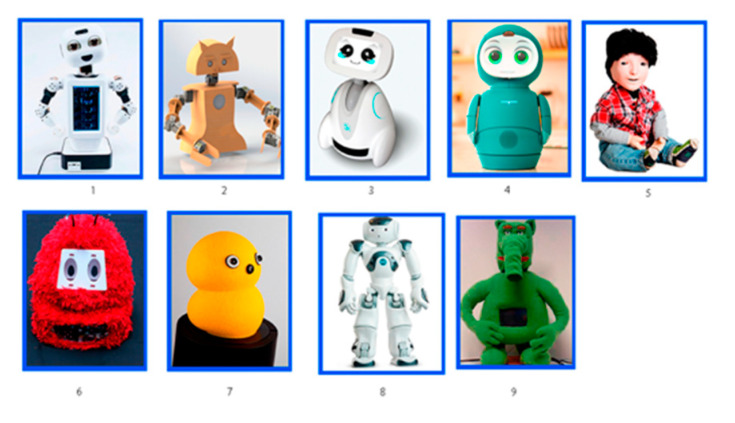
Robot cards.

**Figure 3:**
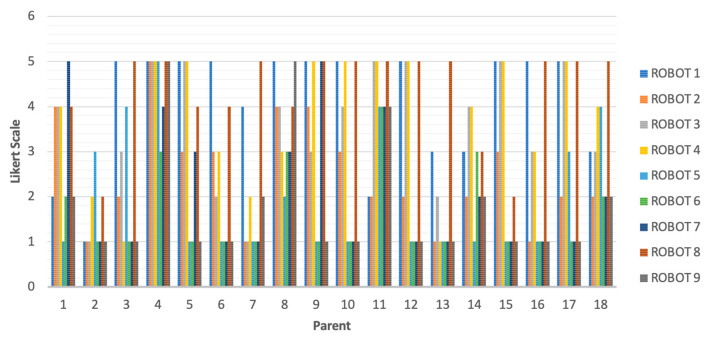
Scores obtained for the appearance of the robots.

Figure 2 shows the assignment given for the appearance of nine robots, evaluated using a five-point Likert scale. Figure 3 shows results obtained, where the highest scoring robot was Robot 8 (78), followed by Robot 1 (73) and Robot 4 (67). Robot 6 and Robot 9, corresponding to the Keepon and Probo robots, respectively, were the ugliest. Robot 1 and 8 were both anthropomorphic and had better appearance scores.

**Figure 4:**
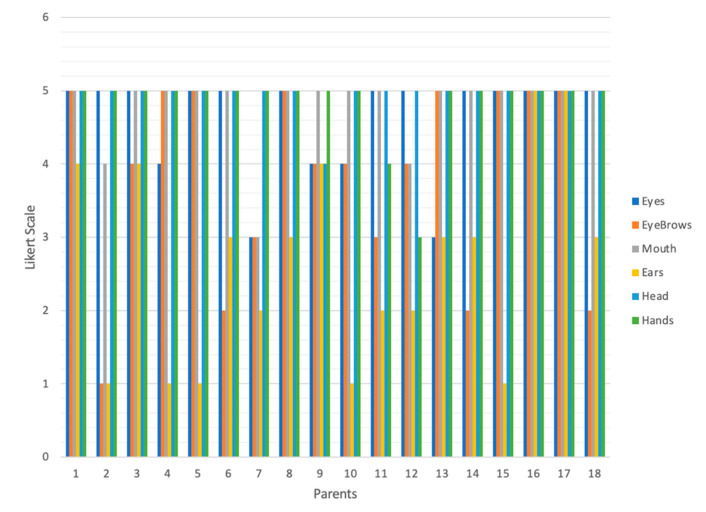
Scores obtained for those body parts essential for a robot to teach an emotion.

Figure 4 shows the assignment given to the body parts essential for a robot to teach an emotion, also evaluated using a five-point Likert scale. The body parts with the highest values were head (89), hands (87), and eyes (83).

Then, the questionnaire was refined and validated with thew support of one psychologist and was administered to the parents and therapists of children with ASD from Colombia. Both instruments were validated by another psychologist, where aspects such as the general objective of the research; the clarity of the variable(s) and category(ies) of analysis; the coherence of the item-variable(s)/category(ies); the item measures’ variable(s)/category(ies); the wording of items; item spelling; instrument presentation; population/sample selection; procedure; assisted consent; and ethical, bioethical, and deontological issues were considered.

Then, new information was collected from seven therapists (five psychologists; one speech therapist; and one special education teacher) and 18 parents of children with ASD. Some sociodemographic characteristics were identified, for example, 86% of therapists had ASD patients in the range of 5 to 10 years, where 43% worked with severity level three patients and 29% with level one and two.

The questions given to and answers provided by the specialists are outlined below.

**Q1: *What types of therapy have been used for children with ASD?***

RQ1: Behavioral therapy, speech therapy/audiology, socio-affective therapy, and ABA therapy.

**Q2: *How do you teach a child with ASD to identify emotions and their physiological responses?***

RQ2: Through games, stories, rounds, videos, examples, and illustrations, where the notion of the theory of mind, symbolic play, imaginative function, and the recognition of one’s own and others’ emotions are developed. Activities should be carried out in different contexts, as changes in behaviors are taken into account according to the child’s space; in this sense, activities can be carried out with materials that allow the child to identify and feel comfortable (this is something that is done frequently) so that the child manages to adapt to the activities and establishes the expected behavior. In addition, role play and PECS sheets may be used.

**Q3: *Have you used technologies in therapy interventions?***

RQ3: 86% answered “yes” and 14% answered “no”.

**Q4: *Imagine therapy with robots—what would be the advantages and disadvantages?***

RQ4: Some of the answers given were:

- Novelty, interaction with the object, and ease of imitation are advantages. Disadvantages include a preference for the machine and inhibition or poor motivation for interactions with peers or others.
- Some advantages could be the process of implementing advanced ICTs in these therapies and the recognition of new strategies by children and their families. Disadvantages could include the poor adaptation of children with ASD and associated visual diagnoses.
- One advantage of having a robot is to be able to utilize the continuous and specific programming of skills on which the child can work and will be constantly exposed to through the robot, without the emotional alterations of a therapist. A potential disadvantage is the issue of predictable environments in a time of crisis.
- One advantage is innovation, while one potential disadvantage is that the children will get used to the robot and their communication with human beings will suffer.

**Q5: *If a person with ASD interacts with a robot, what kind of physical communication gestures should the robot make?***

RQ5: Some answers were:

- Make gestures approximating signs for greeting, requesting, and pointing.
- The robot should be clear in its tone of voice, movements, and sounds.
- Facial expressions.
- Have understandable facial expressions, straight posture.
- Imitation of all kinds.

**Q6: *What kind of affective tactile communication should the robot be able to feel, and what parts of its body should be sensitive to touch?***

RQ6: Hands, face, and head.

**Q7: *What emotions should the robot express?***

RQ7: Basic emotions such as joy, sadness, and anger, as well as complex emotions such as pride, empathy, and embarrassment.

***Q8: What might a robot look like (e.g., animal, humanoid, large, small, etc.)?***

*RQ8: All answers were humanoid.*

***Q9: What should a robot used in therapy for children with ASD not have?***

RQ9: Some answers were:

- Robotic voice.
- Loose or pointed metal parts.
- Loud or unexpected sounds.
- Sharp artefacts, loud sounds, loud colors, and glass or transparent panels, as they would be distracting. The off button should also be hidden.
- No robotic verbal language, but a “normal” voice.

**Q10: *Some robots (Figure 2) are shown so that you can assign a score. How would you rate their appearance?***

RQ10: Robot 1 (average 2.91); Robot 2 (2.29); Robot 3 (3.43); Robot 4 (3.67); Robot 5 (2.00); Robot 6 (1.86); Robot 7 (2.43); Robot 8 (3.00); Robot 9 (2.00).

**Q11: *Of the robots you have just seen, which one did you like the most, and what improvements would you make to its appearance?***

RQ11: Eyes (average 4.71); eyebrows (4.43); mouth (4.86); and ears (2.71).

**Q12: *Which parts of the body do you think are most important for teaching a child with ASD about emotions?***

RQ12: Head (average 4.57) and hands (5.0). Another answer suggested a heart that beats and arms.

Then, we obtained the answers of 18 parents of children with ASD aged 10–15 years (50%); 3–5 years (17%); or 5–10 years (33%).

Some of the questions formulated and answers provided are outlined below.

**Q1: *Select the type of therapy that has been implemented for your child with ASD?***

RQ1: Behavior-based therapy (5); speech therapy (6); occupational therapy (4); socio-affective therapy (1); or others (2)

**Q2: *Some robots (Figure 2) are shown so that you can assign a score. How would you rate their appearance?***

RQ2: Robot 1 (3.94); Robot 2 (2.11); Robot 3 (2.76); Robot 4 (3.61); Robot 5 (2.56); Robot 6 (1.72); Robot 7 (2.00); Robot 8 (3.67); Robot 9 (2.22).

**Q3: *Of the robots you have just seen, which one did you like the most and what improvements would you make to its appearance?***

RQ3: Some answers were: The real looking one of a boy, or I would make boy and girl versions, highlighting facial features.

**Q4: *Of the robots you have just seen, which one did you like the most, and what improvements would you make to its appearance?***

RQ4 Eyes (average 4.56); eyebrows (3.72); mouth (4.56); and ears (3.11).

**Q5: *Which parts of the body do you think are most important for teaching a child with ASD about emotions?***

RQ5: Head (4.35) and hands (4.53). Another answer suggested arms.

**Figure 5:**
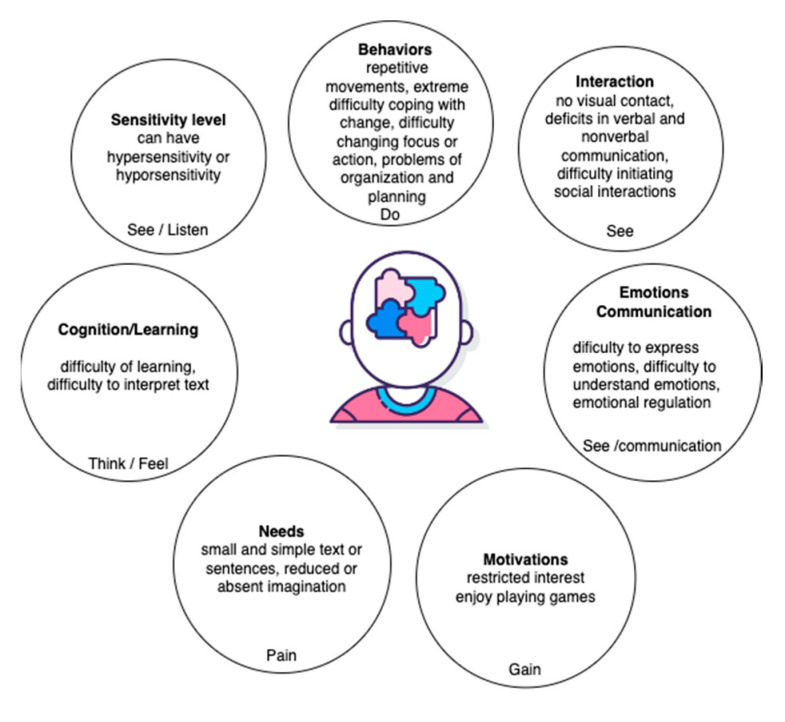
Empathy map for children with autism.

According to the DSM-V, the information collected from the parents of children with ASD and experts in ASD was used to make an empathy map for autism, shown in Figure 5, with components related to “listen”, “think and feel”, and “say”. This map can help to provide information about children with ASD for designers. In addition, many robotic designers are unprepared to design robots for this audience.

Finally, we carried out a comparative analysis of three social robots designed for children with ASD (see Table 1).

### 4.2. Ethical Considerations

The interaction is carried out via facial and body expressions, voice, and other visual elements. The height of the robot can be adjusted. The robot must allow for data to be registered during each interaction. It is suggested that the color red be associated mainly with negative feelings during the social interaction. The robot must be autonomous in its interactions. The robot must be programmed to engage in a wide range of social behaviors.

Some ethical considerations were mentioned in the questionnaires filled out by the specialists and parents of children with ASD:

- The robot should have a humanoid appearance.
- Emotions should be conveyed using facial expressions, body expressions, and hands.
- The robot should allow for the adjustment of gender (i.e., girl robot or boy robot).
- The robot’s voice should not be robotized.
- The robot should express basic emotions as well as some complex emotions, such as pride, empathy, and embarrassment.

### 4.3. Aspects of Design

**Figure 6:**
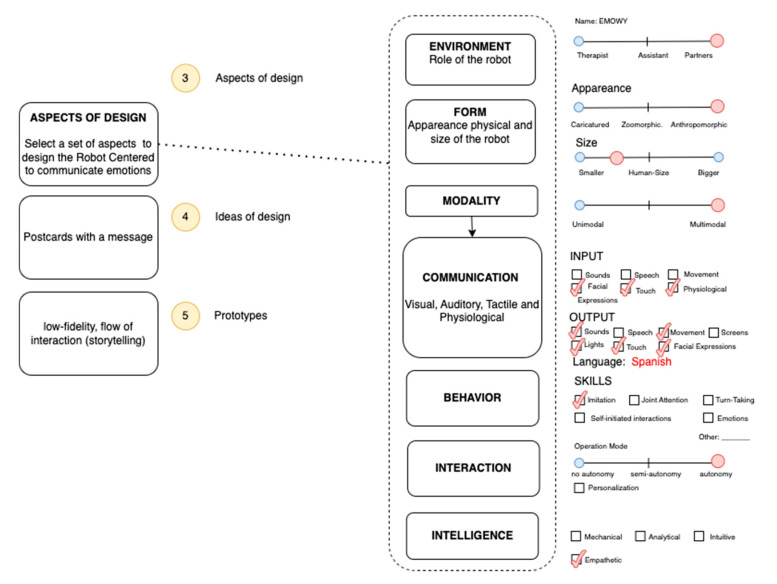
Selecting aspects of design to be considered according to the collected information.

In this stage, we considered some guidelines proposed by [17,74], such as technical, mechanical, and manufacturing features. Figure 6 shows aspects of design to be considered in the study case.

### 4.4. Design Ideas

**Figure 7:**
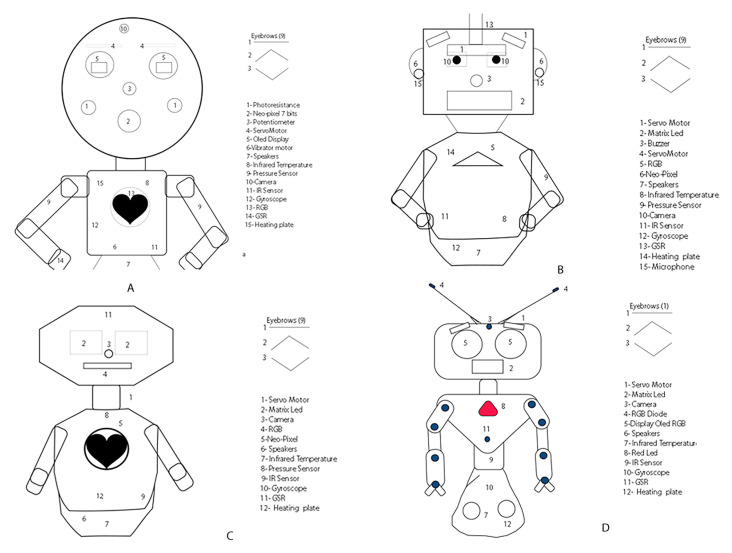
Graphical representation of the design (wireframes), which were proposed four prototypes. (**A**) prototype 1; (**B**) prototype 2, (**C**) prototype 3; (**D**) prototype 4.

Figure 7 shows some low-fidelity design ideas. According to the needs identified, it was taken into account that the robot must have a humanoid appearance; therefore, the facial characteristics considered are eyebrows, nose, cheeks, ears, mouth, and eyes. In Figure 7A, the eyes are two low-cost OLED displays that simulate blinking. One RGB camera is also considered to recognize objects and people’s faces. Meanwhile, in Figure 7B, the eyes are static; however, the robot has eyebrows and eyelids, whose mechanics use servo-motors. In the robot shown in Figure 7C, the eyes are LED matrix size 8 bits. In two prototypes, the nose is considered as a potentiometer (Figure 7A) or a buzzer (Figure 7B). Another facial feature considered is the mouth, represented by an RGB matrix in the three prototypes.

In Figure 7B, the presented prototype has ears, which are two NEO-pixels size 8 bits located on each side of the head that light up whenever the robot is listening to the user or expressing a negative emotion. Finally, the design in Figure 7A includes cheeks, aiming to motivate the child to approach the robot and touch its cheeks.

According to answers obtained in the questionnaire given to experts and parents, the robot’s appearance is essential. Therefore, we selected the prototype shown in Figure 7B after modifying the form of the face. The expressiveness of emotions is fundamental to teaching this concept to children with ASD. Therefore, a face that can express basic emotions such as anger, fear, happiness, sadness, surprise, and disgust, as proposed by Ekman [74], was designed.

On the other hand, physiological inputs and outputs were also considered in the design aspects. Thermal expression refers to emotion-associated changes in body temperature; it is an involuntary response [75]. Typically, the change in body temperature is activated by negative emotional states. The use of thermal expression in social robots has benefits such as

- Unaltered robot shape.
- Privacy (only the person who is interacting physically with the robot can feel the thermal stimulus).
- People who do not interact physically with the robot cannot perceive the thermal stimulus.

The output temperature can be localized in the torso of the robot, considered here as a heating plate. However, as an emotional expression, the temperature requires a higher degree of abstraction to be understood as an emotional state. For capturing physiological inputs such as galvanic sensor response, temperature, and heart rate, the child must use a wearable device such as a bracelet or smartwatch as an external accessory.

### 4.5. Prototype

**Figure 8:**
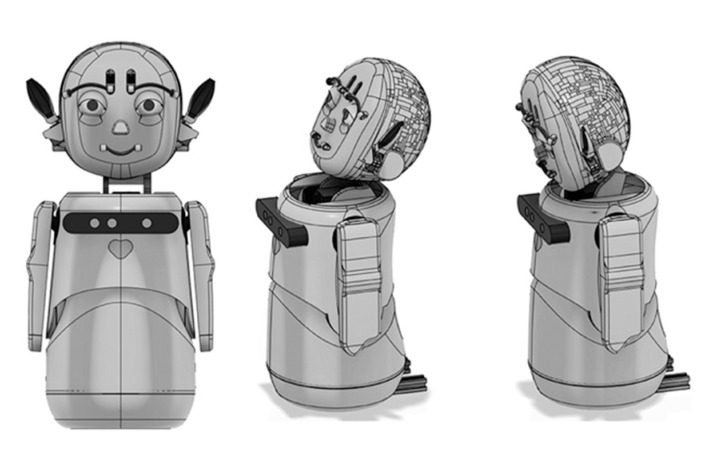
3D models of the proposed robot.

After the conception of some design ideas, we selected a set of inputs and outputs and proposed a final version of the social robot. Figure 8 shows a 3D model of the social robot with a humanoid appearance; the robot has a child-like appearance because of its role as a partner. Body expressions were also considered, including movements of the head, neck, arms, torso, and hands.

**Figure 9:**
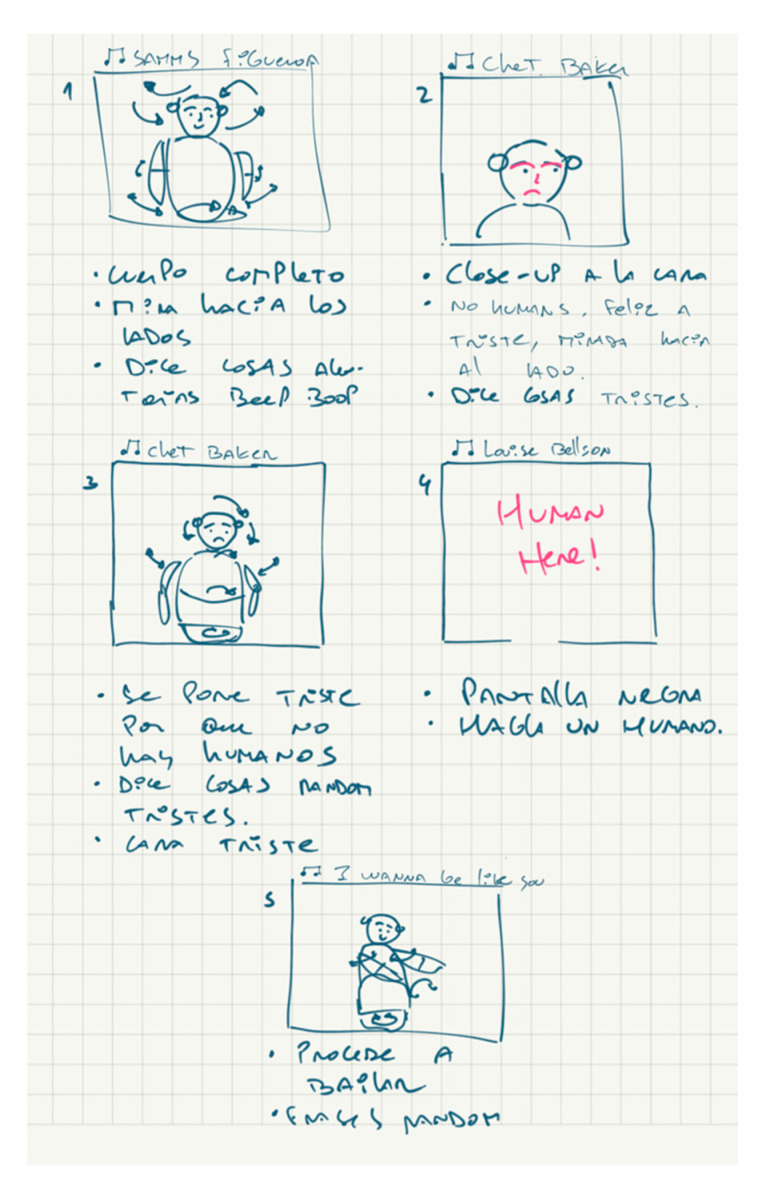
Sketches of five scenarios to demonstrate and emulate the use of the mechanical features of the social robot to communicate emotions.

Three-dimensional models were designed and printed to implement the input and output hardware of the robot. To demonstrate that the robot could use all its mechanical features to emulate and express emotional states, a script was designed consisting of a history of a tiny robot that is craving human interaction (Figure 9); the robot cannot feel sad, but when a human talks to it, it becomes happier and starts to dance.

**Figure 10:**
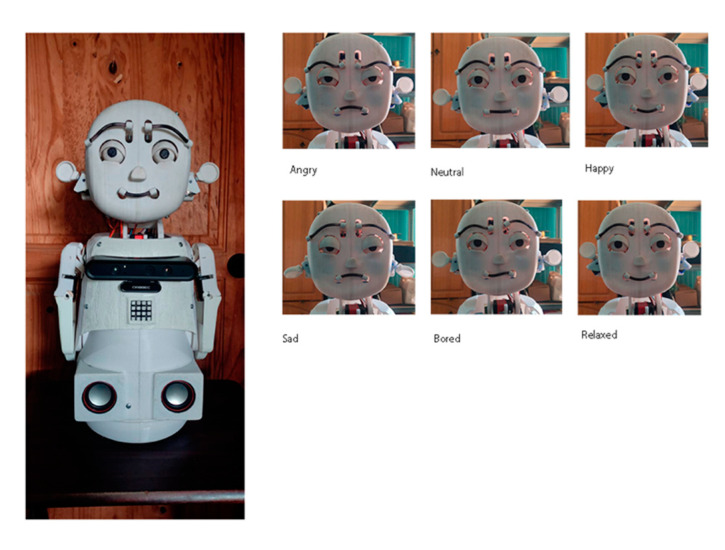
Facial expressions of the social robot.

**Figure 11:**
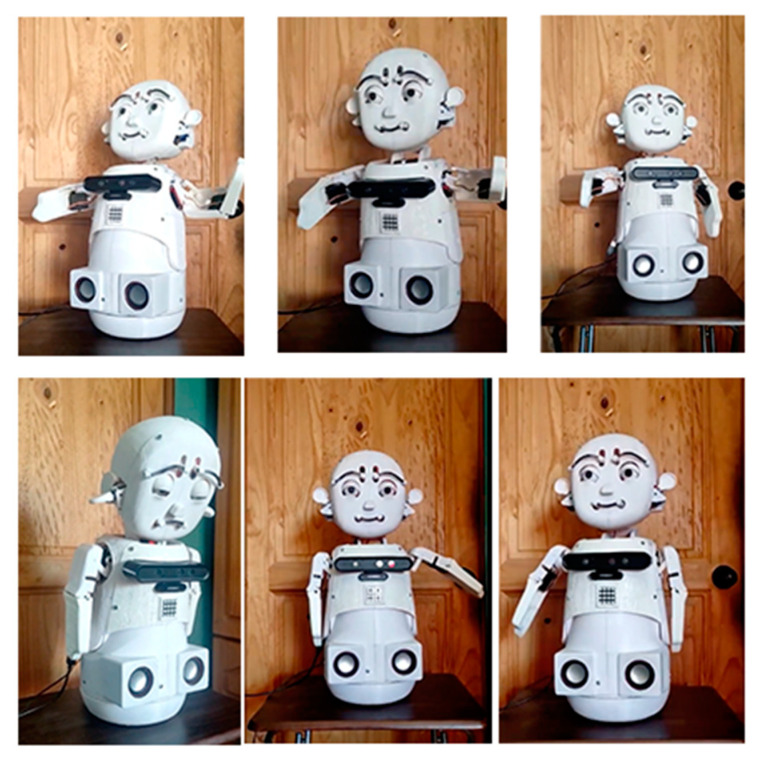
Body expressions of social robot.

Figure 10 and Figure 11 show some facial and body expressions of the robot, which were implemented to evaluate the factors of the robot.

Once the basic functionalities were implemented, the UEQ questionnaire [76] was applied to evaluate the following scales:

- Attractiveness: the impression of the product.
- Perspicuity: the ease of use of the product.
- Stimulation: how engaging the product is.
- Novelty: the novelty of the product.

The UEQ is grouped into six scales, where each item is evaluated on a seven-point Likert scale. The answers are scaled from −3 (fully agree with the negative term) to +3 (fully agree with the positive term). Half of the items start with the positive term, while the others start with the negative term. *Attractiveness* is a pure valence dimension (emotional reaction on a pure acceptance/rejection dimension), while *stimulation* and* novelty* are hedonic quality aspects related to pleasure or fun while using the product. This questionnaire is accessible at www.ueq-online.org in several languages.

**Figure 12:**
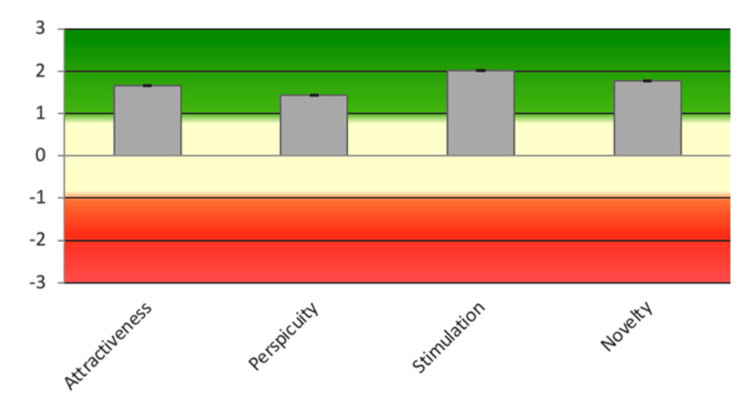
Results obtained from the UEQ questionnaire.

A group of 12 experts in areas including psychology (one), human–computer interaction (HCI) (seven), and human–robot interaction (HRI) (two), and parents of children with ASD (2) completed the UEQ. Figure 12 and Table 2 show the results obtained, which were satisfactory.

The scales of the UEQ can be grouped into pragmatic quality (perspicuity, efficiency, and dependability) and hedonic quality (stimulation and originality). Pragmatic quality describes task-related quality aspects, while hedonic quality refers to non-task-related quality aspects. Table 3 shows the calculated mean of the three aspects. The results were shown to be very satisfactory overall based on hedonic quality.

## 5. Discussion

Reinforcement of the communication of emotions is essential in children with ASD due to their deficits in perceiving, understanding, and expressing emotions. Therefore, the appearance of social robots can be essential in communicating emotion and motivating children with ASD, especially if the robot plays the partner role. Robins et al. [77] presented a study about the impact of a social robot’s appearance on interaction in children with ASD. It was found that the robot should not be too human-like because it could lose the child’s interest. In addition, it was suggested that complex facial expressions be avoided to enhance simplicity. Therefore, the prototype was created to be humanoid, including facial features such as eyes, eyebrows, a mouth, eyelids, and a nose. Another important aspect is size, since the robot must establish eye contact with the child. According to [78], children tend to show greater stimulation in response to robots with pet-like or cartoon-like features. Humanoid robots may be preferred to teach emotional skills. Kozima et al. [79] mentioned that robots with overly mechanized appearances may also not yield the best results, since too many mechanical parts are exposed.

In the data collection conducted to investigate the characteristics of children with ASD, it was determined that reading and interpreting facial expressions and body language [80] is challenging for them. This is because interacting with others can involve excessive sensory stimulation, leading to distress. Human–robot interactions are different, as robots can be programmed to show basic emotions. In turn, the robot can communicate with the child through simple tasks in such a way that it can avoid sensory overload [81]. In addition, communicating emotion through various channels can cause an overload. Therefore, it is necessary to establish which channels are the most appropriate for expressing emotions so that the child can understand the robot’s emotional state.

On the other hand, children with ASD tend to express hostile rather than positive emotional states, and very often, these negative emotional states can influence specific physiological responses, which they may be somewhat unaware of. Thus, acting out physiological responses can support them in becoming aware of this.

The design of social robots can follow a UCD approach, which involves applying a set of methods and techniques to investigate the user and the context of use. This facilitates the creation of mechanical interfaces according to the user’s needs, especially when the target audience consists of children; this means designing technology for the needs, abilities, and expectations of children with ASD.

On the other hand, the design of a social robot can also follow an inspirational approach according to the three aspects proposed by Norman [82], i.e., visceral, behavioral, and reflective, which can be related to appearance, functionality, and symbolism.

## 6. Conclusions and Future Work

In the literature reviewed, studies on the designing of a social robot for children with autism were rare; many of them focused on a participatory design but did not include aspects that would allow for the structuring of how a social robot should be designed, such as its appearance, size, age, gender, interaction type, and intelligence level.

The design of emotional communication for a social robot includes emotional intelligence. Therefore, the robot must perceive, understand, and express emotions. In children with ASD, sensory channels are more significant, and these individuals also process sensory information differently from children without ASD. However, the question remains of how the robot can empathize with the user by providing more appropriate and adaptive responses during the interaction.

Cano et al. [83] suggest that emotional communication models in social robots for children with ASD must consider the following aspects:

1. The appearance of the physical robot, which can help it to empathize with the child.
2. Communication channels (verbal and non-verbal cues) to express an appropriate emotional state.
3. Types of sensors to perceive emotions and techniques to recognize a target’s emotion.
4. Theories of psychology that can support the learning of socio-emotional skills.
5. Autonomous empathic behavior responses.

Several studies have attempted to explore multimodal emotion by combining two modalities; however, comparing more than three modalities is rare. Therefore, there is a need to develop a comprehensive perspective on how different combinations of modalities (gaze, gesture, tactile, and physiologic) contribute to the perception of emotions during an interaction. Several studies have found that the designs of robots for ASD are oriented towards visual and auditory cues. Although they are usually very visual, they are not usually very auditory, and more work needs to be carried out with respect to the tactile part. This aspect was identified in the design path in the first stage, which consists of analyzing information related to autism and therapy.

On the other hand, the case study that was applied using the design path showed that, by following the user-centered design approach, a mechanical interface could be obtained according to the user’s needs. Therefore, it is vital to involve the end-users; in this case study, the methods were applied to parents of children with ASD or specialists, because children with ASD have problems with external communication. In the future, a part of the participatory design could be applied to them, where they can help design the robot’s appearance but not its functionalities.

One limitation of this work was that we found few studies on the design of social robots for Latin American culture. We were not able to interact with children with ASD to learn about how they perceive or imagine a robot and how they imagine the functionalities that a robot could have. In addition, to evaluate the acceptability of the social robot among children with ASD.

This proposal is limited, as it serves as a support to consider aspects that should be taken into account when designing a social robot. However, it does not indicate, for example, how to design a computational model that can provide the social robot with emotional intelligence. Moreover, the design of these computational models can embed theories of psychology and therapy, among others; however, the literature is very scarce with respect to the most adequate theories to be used in the design of the computational model, which gives intelligence to the robot.

In future work, this tool must be applied to the design of more social robots. In addition, the impact and level of priority of the design variables that were included in the design path should be assessed. Finally, a set of recommendations for the design path should be defined.

## Figures and Tables

**Table 1:** Comparative analysis of social robots for children with ASD.

Hardware Aspects	Moxie	Nao	Kaspar
Appearance	Humanoid	Humanoid	Humanoid
Height	38.7 cm	58 cm	56 cm
Gender	-	-	Boy
Biped	False	True	False
Facial expressions	Uses display representing face features such as eyes, mouth, and eyelids	Eyes change of color.	Eyes, mouth, eyelids
Open programming for researchers	False	True	False
Movements	Head, torso, arms, neck and hands	Head, arms, hands, pelvis, legs	Head, neck, arms, torso
Total servos (actuators)	9	25	22
Sensors	Four microphones, one camera 5MP	OmniVision cameras, inertial sensor, sonar range finder, infrared sensors, tactile sensors, pressure sensors; microphones	Cameras in eyes; force sensors
Response multimodal	Voice and visual	Voice, tactile, and visual	Visual, tactile, and voice
Language	English	English	English
Country	USA	France	United Kingdom

**Table 2:** UEQ Scales (mean and variance).

Scale	Mean	Variance
Attractiveness	1.652	0.23
Perspicuity	1.434	0.11
Stimulation	2.023	0.11
Novelty	1.773	0.23

**Table 3:** Pragmatic and hedonic quality.

Scale	Hedonic Quality
Attractiveness	1.65
Pragmatic Quality	1.23
Hedonic Quality	1.90

## Data Availability

Not applicable.
